# Switching Stable Kidney Transplant Recipients to a Generic Tacrolimus Is Feasible and Safe, but It Must Be Monitored

**DOI:** 10.1155/2017/5646858

**Published:** 2017-01-26

**Authors:** Fernando González, René López, Elizabeth Arriagada, René Carrasco, Natalia Gallardo, Eduardo Lorca

**Affiliations:** ^1^Department of Nephrology, Hospital del Salvador, Santiago de Chile, Chile; ^2^Faculty of Medicine, Universidad de Chile, Santiago de Chile, Chile; ^3^Faculty of Medicine, Clínica Alemana, Universidad del Desarrollo, Santiago de Chile, Chile

## Abstract

*Background*. Tacrolimus is the primary immunosuppressive drug used in kidney transplant patients. Replacing brand name products with generics is a controversial issue that we studied after a Chilean Ministry of Health mandate to implement such a switch.* Methods*. Forty-one stable Prograf (Astellas) receiving kidney transplant patients were switched to a generic tacrolimus (Sandoz) in a 1 : 1 dose ratio and were followed up for up to 8 months. All other drugs were maintained as per normal practice.* Results*. Neither tacrolimus doses nor their trough blood levels changed significantly after the switch, but serum creatinine did: 1.62 ± 0.90 versus 1.75 ± 0.92 mg/dL (*p* < 0.001). At the same time, five graft biopsies were performed, and two of them showed cellular acute rejection. There were nine infectious episodes treated satisfactorily with proper therapies. No patient or graft was lost during the follow-up time period.* Conclusion*. Switching from brand name tacrolimus to a generic tacrolimus (Sandoz) is feasible and appears to be safe, but it must be monitored carefully by treating physicians.

## 1. Introduction

Tacrolimus is the primary immunosuppressant used in solid organ transplant patients [1, 2]. It has a narrow therapeutic index, and several studies have demonstrated that therapeutic drug monitoring provides information of predictive value for managing the risk of concentration-related rejection and toxicities [3–5]. Brand name tacrolimus (Prograf™, Astellas Pharma US, Deerfield, IL) lost patent protection in 2008, and the first generic tacrolimus product was Food and Drugs Administration (FDA) approved in 2009 (Sandoz, Holzkirchen, Germany) [6].

Generic products must document comparable bioavailability with their innovator counterpart as dictated by regulatory agencies, like FDA or EMA. These bioequivalence studies are normally performed in healthy volunteers after a single dose of the drug in a crossover design [7]. Observations in normal healthy volunteers may not necessarily represent what is likely to happen in solid organ transplant recipients, possibly because they do have comorbidities, such as gastrointestinal motility disorders or use of concomitant medications that could affect pharmacokinetics of drug being studied [8, 9]. From this point of view, it is important to have pharmacokinetic data derived from transplanted patient to guide physicians on how to use generic products safely [10–12].

Alloway et al., in a prospective, multicenter, open-label, randomized crossover study undertaken with the objective of comparing the steady-state pharmacokinetics of a generic tacrolimus (Sandoz) versus the originator drug (Prograf) in stable kidney transplant patients, observed a similar pharmacokinetic profile for both products according to US FDA and European Medicines Agency guidelines (EMA) [13].

The use of generic medications is widespread and represents a viable cost-saving opportunity in the face of rising health care cost [13, 14]. While an FDA approved generic tacrolimus is expected to be efficacious, a clinically relevant risk of altered drug exposure may exist when converting patients from one product to another. In this publication, we report the experience of conversion from brand tacrolimus (Astellas) to a generic formulation (Sandoz) in reference to tacrolimus dose and blood levels, graft function and acute graft rejection episodes, and infections in Chilean kidney transplant recipients.

## 2. Materials and Methods

Following a Chilean Ministry of Health mandate, all patients receiving immunosuppressants for kidney transplantation in public hospitals must be switched to generic products. This allowed us to conduct this single-center, prospective, nonrandomized study based on clinical and laboratory information registered in clinical charts of the kidney transplant recipients. The identity of the patients was guarded during collection and analysis of research data. Our Institutional Review Board waived us from obtaining an informed consent form to switch from the innovative to the generic drug, but all patients consented to their data to be included in the database.

We included all adult patients with more than three months after transplantation, with stable preswitch tacrolimus dose and blood levels for at least 4 weeks and at least three blood tacrolimus and serum creatinine blood levels before and after the switch. The tacrolimus conversion was conducted between September and October 2012 in a 1 : 1 dose ratio as previously described by Momper et al. [15]. The doses of coprescribed medications known to interfere with the metabolism of tacrolimus were maintained stable. All drugs were dispensed in the hospital.

All patients were instructed to take tacrolimus doses at specified times to ensure accurate determination of trough blood concentrations. After the conversion, the dose of generic tacrolimus was adjusted at the discretion of the treating physician to maintain trough concentrations within the therapeutic range. All patients were followed up for up to eight months. All other immunosuppressive drugs were maintained as usual (mycophenolic acid derivatives or azathioprine and steroids). A chemiluminescent microparticle immunoassay (CMIA) was used for the quantitative determination of tacrolimus.

Paired Student's* t*-test was used to analyze the data. The threshold of statistical significance was set at 5%. No adjustments were made for multiple comparisons.

## 3. Results

There were 57 tacrolimus users. Forty-one (61% male, mean age 38 years old) complied all inclusion/exclusion criteria; from them, a total of 246 tacrolimus trough concentrations were included in the analysis. Neither pre- and postconversion tacrolimus blood trough concentrations (8.0 ± 2.2 vs 7.4 ± 1.6 ng/mL; *p* = 0.354), tacrolimus daily dose (3.88 ± 1.98 versus 4.11 ± 2.05 mg/d; *p* = 0.308), nor weight normalized daily doses (0.052 ± 0.023 versus 0.055 ± 0.033 mg/Kg/d; *p* = 0.600) differed significantly. Also, tacrolimus blood levels normalized to daily dose (2.16  ±  1.40 vs 2.09  ±  1.41 ng/mL/mg; *p* = 0.906) and tacrolimus blood levels normalized to daily dose adjusted by body weight (138.6 ± 104.8 vs 143.9 ± 96.7 ng/mL/mg/kg; *p* = 0.207) did not change significantly. Nevertheless, preconversion serum creatinine was statistically lower than the postconversion: 1.62  ±  0.90 versus 1.75  ±  0.92 mg/dL (*p* < 0.001) (Table 1).

At follow-up, five patients were biopsied because of an increased serum creatinine (12.2%): two had an acute cellular rejection episode (Banff Ib and IIa; at day 45 and month 8, resp.), two had interstitial fibrosis and tubular atrophy, and the other had findings that supported a BK virus infection. Nine infectious events were observed: Six urinary tract infections, one community acquired pneumonia, one herpes zoster, and one pityriasis versicolor (Table 1). No patient discontinued treatment or follow-up, and no graft loss or death was observed.

## 4. Discussion

Considering that this is neither a controlled clinical nor a cohort comparative trial, some clinical teachings can be recognized when facing the necessity to switch narrow therapeutic window drugs in transplant patient population. For example, it is obvious that acute rejection episodes can occur if patients are under immunosuppressed or infectious adverse events can occur if patients are over immunosuppressed; but if clinicians are conscious of that, they can mitigate those hazards by seeing patients and controlling the blood drug levels more frequently. We, indeed, observed a couple of graft rejection episodes and some, but not severe, infectious episodes that are also frequently observed in adult internal medicine patients.

In spite of not observing tacrolimus dose requirement changes or in their trough blood levels after the switch from the innovative to the generic drug, allografts function, indeed, appeared to deteriorate as serum creatinine increased marginally approximately 0.13 mg/dL (*p* < 0.001). The clinical implication of these findings is not clear because it can, even, be interpreted either as a random laboratory result variation comparable to its biological variation [16] or representing the resultant from the natural course of the kidney transplantation or a statistical consequence from the acute graft rejection episodes observed. Nevertheless this post switch creatinine increase merits observation because it is not commonly observed in other settings without the necessity of changing immunosuppressive drugs [17].

Another clinical teaching from this experience is that immunosuppressant drug switches, even to a well validated generic, as required by FDA or EMA, must be monitored. We observed two cellular acute rejection episodes at 1.5 and 8 months of follow-up, and, in spite of not having a preswitch observation control period and the possibility that both rejections could be attributed to other clinical factors different from the generic tacrolimus, the message is that treating transplant physicians must know what trademark drug products their patients are consuming in order to estimate the probability of adverse events or adverse outcomes.

Infectious episodes observed were of no clinical significance, but, once again, there is a necessity to monitor the patients whenever there is a change in their immunosuppression.

Our results are in concordance with others. Momper et al. [15], in 55 kidney transplant patients followed for 14–90 days before and after the generic conversion, observed a lower concentration/dose ratio and a small drop in tacrolimus concentrations without appreciable change in kidney function or acute rejection rate. McDevitt-Potter et al. [18] observed, in 37 kidney transplant patients, that dose requirement and trough levels were similar between brand and generic tacrolimus and that generic substitution allows for savings. In Chile, Müller et al. [19], in 17 kidney transplants followed up for 7.6 months, reported that generic tacrolimus yielded effective and safe immunosuppression in terms of mortality, biopsy-proven acute rejection, and graft loss with a low incidence of adverse effects. Our findings also reinforce the stability and usefulness of the generic tacrolimus.

We recognize that our study is not a formal clinical trial as others are [15, 18, 19]. But, at the same time, our experience could be considered more representative of the real world clinical practice, where the majority of busy transplant centers work every day facing patients, administrative, and regulatory pressure. Our observations could contribute to assure transplant physicians that good quality generic immunosuppressant drugs show acceptable safety profiles.

In conclusion, converting stable kidney transplant recipients from Prograf to an FDA and EMA approved generic tacrolimus is feasible and appears to be safe, but, nevertheless, it is suggested that close monitoring of patients and clinical monitoring of the graft function be implemented and maintained in time.

## Figures and Tables

**Table 1:** Creatinine, tacrolimus blood levels, and tacrolimus doses before and after the conversion in all patients and selected subgroups (those who maintained stable or deteriorated allograft function and those who suffered infections or underwent biopsies).

	Baseline			Postconversion			Between periods *p* values		
	Creatinine mg/dL	Tacrolimus		Creatinine mg/dL	Tacrolimus		Creatinine	Tacrolimus	
		Blood level ng/mL	Dose mg		Blood level ng/mL	Dose mg		Blood levels	Dose
All patients (*n* = 41)	1,62 ± 0,90	8.0 ± 2,2	3,9 ± 2.0	1,75 ± 0,92	7,4 ± 1,6	4,1 ± 2,1	<0,001	0,354	0,308

Stable creatinine (*n* = 13)	1,75 ± 0,38	8,2 ± 2,5	3,9 ± 1,7	1,6 ± 0,4	7,2 ± 1,6	4,7 ± 2,1	0,003	0,452	0,116
Increased creatinine (*n* = 28)	1,53 ± 0,74	8,2 ± 2,0	4,3 ± 2,2	1,9 ± 1,0	7,6 ± 2,5	4,4 ± 2,1	<0,001	0,265	0,268

Between groups (*p* = )	0,265	0,94	0,557	0,374	0,537	0,617			

Biopsied patients (*n* = 5)	1,78 ± 0,34	8,5 ± 1,3	5,5 ± 3,4	2.0 ± 0,4	7,1 ± 1,8	5,4 ± 2,4	0,120	0,340	0,721
Infected patients (*n* = 9)	1,38 ± 0,52	9,0 ± 1,8	3,8 ± 1,2	1,62 ± 0,58	8,6 ± 3,6	4,2 ± 1,7	0,194	0,775	0,404

Biopsied versus not biopsied	0,636	0,617	0,245	0,68	0,858	0,361			
Infected versus not infected	0,314	0,168	0,706	0,595	0,085	0,727			

## Additional Points

*Short Summary*. Switching kidney transplant patients from brand name to generic immunosuppressant is controversial. There is scarce data from Hispanic population. After a ministry of health mandate to begin prescribing generics we report a positive experience, but, at the same time, we caution the transplant community to perform a carefully monitored switch to generics.
